# Participation of private providers in the National TB Programme in South India

**DOI:** 10.5588/pha.23.0032

**Published:** 2023-12-07

**Authors:** A. D. Meundi, J. H. Richardus

**Affiliations:** 1 Department of Community Medicine, Dr Chandramma Dayananda Sagar Institute of Medical Education and Research, Dayananda Sagar University, Bengaluru, India; 2 Department of Public Health, Erasmus MC, University Medical Center Rotterdam, The Netherlands

**Keywords:** barriers, facilitators, involvement, NTEP

## Abstract

**SETTING:**

India has the highest number of new TB cases worldwide. The participation of private providers (PPs) in the National TB Elimination Programme (NTEP) has remained suboptimal.

**OBJECTIVE:**

To explore the experiences, barriers and facilitators about their participation in the NTEP as perceived by PPs working in varied settings.

**DESIGN:**

Focus group discussions and in-depth interviews were used to engage PPs to obtain their views on participation in the NTEP. Framework and thematic content analysis was used to analyse qualitative data.

**RESULTS:**

Non-availability of a comprehensive range of diagnostics and lack of flexibility in the NTEP were barriers to participation in NTEP. PPs were predisposed to think that NTEP was for those who could not afford to purchase medications. Attitudes and previous experiences with NTEP made them sceptical about the NTEP regimen. Although more frequent interactions were sought with NTEP, some bitterness about previous interactions was perceived.

**CONCLUSION:**

Challenges identified by PPs for the NTEP include improvement of the quality of TB care, especially at the lower levels of care, availability of a comprehensive range of diagnostics, being friendly to PPs and patients, more frequent interactions with PPs, and more caring conversations with patients at NTEP centres.

## Abstract (fr)

**CONTEXTE:**

L’Inde est le pays qui compte le plus grand nombre de nouveaux cas de TB dans le monde. La participation des prestataires privés (PP) au programme national d’élimination de la TB (NTEP) est restée sous-optimale.

**OBJECTIF:**

Explorer les expériences, les obstacles et les facilitateurs de leur participation au NTEP, tels qu’ils sont perçus par les prestataires privés travaillant dans des contextes variés.

**MÉTHODE:**

Des discussions de groupe et des entretiens approfondis ont été organisés afin d’obtenir le point de vue des professionnels de la santé sur leur participation au NTEP. L’analyse de cadre et de contenu thématique a été utilisée pour analyser les données qualitatives.

**RÉSULTATS:**

La non-disponibilité d’une gamme complète de diagnostics et le manque de flexibilité du NTEP ont constitué des obstacles à la participation au NTEP. Les PP étaient prédisposés à penser que le NTEP était destiné à ceux qui n’avaient pas les moyens d’acheter des médicaments. Les attitudes et les expériences antérieures avec le NTEP les ont rendus sceptiques quant au régime du NTEP. Bien qu’ils aient souhaité des interactions plus fréquentes avec le NTEP, ils ont ressenti une certaine amertume à l’égard des interactions antérieures.

**CONCLUSION:**

Les défis identifiés par les PP pour le NTEP comprennent l’amélioration de la qualité des soins antituberculeux, en particulier aux niveaux inférieurs de soins, la disponibilité d’une gamme complète de diagnostics, l’amabilité envers les PP et les patients, des interactions plus fréquentes avec les PP et des conversations plus attentionnées avec les patients dans les centres du NTEP.

Globally, TB was the 13^th^ leading cause of death and second leading infectious killer after COVID-19 in 2020. In 2020, 30 high-burden countries accounted for 86% of new cases of TB, with India leading the count.^1^ The average contribution of private providers (PPs) to total TB notifications between 2011 and 2016 in Bangalore City, India, was 20%.^2^ In 2021, a total of 2.1 million TB cases were notified in India, of which 68% were from the government sector and 32% were from the private sector, when in fact, about 50% of patients had consulted PPs.^3^ Between 2019 and 2021, the prevalence:notification ratio was 2.84, suggesting under-notification or that a substantial number of cases had been missed.^4^ With the National TB Elimination Programme (NTEP) envisaging a nationwide reduction of TB incidence by 80% by 2025 compared to 2015, involvement of PPs has risen in priority. The National Strategic Plan for TB Elimination in India includes private sector engagement as one of the four thrust areas.^5^ Studies have shown that half of suspected TB cases had visited a PP before approaching the government healthcare system.^6^,^7^ Only one third of these TB presumptives who went to a qualified provider in the private sector went on to a government centre after the first consultation, the rest taking up to six consultations before reaching a government centre.^8^

The experiences of PPs in their interaction with the NTEP have been shown to influence the participation of PPs in the programme,^9^,^10^ and efforts to engage PPs have demonstrated improved contribution from the private sector in India.^11^ The present study aims to explore the experiences of, barriers to and facilitators of PP participation in the NTEP as perceived by PPs working in varied settings.

## METHODS

Qualitative methods, i.e., focus group discussions (FGDs) and in-depth interviews (IDIs), were used to engage PPs to obtain their views on participation in the NTEP. Participants of the present study were specialist faculty of medical colleges, private specialists and general practitioners. All were employed and/or practicing in Bangalore City under the city’s municipal authority, Bruhat Bengaluru Mahanagara Palike (BBMP). Eight FGDs were conducted with faculty of medical colleges, including specialists from the Paediatrics, Internal Medicine and Pulmonology Departments. Three medical colleges were covered. Each FGD group consisted of a single speciality and participants belonging to all academic cadres (senior residents, assistants, and associate and full professors). Twenty-four IDIs were conducted with private specialists, which included 6 paediatricians, 6 internal medicine specialists and 6 pulmonologists (all outside medical colleges), 4 IDIs with general practitioners (with only a MBBS [Bachelor of Medicine and Bachelor of Surgery] degree) and 2 IDIs with hospital administrators (Table 1). Purposive sampling was used for the FGDs and snowball sampling for the IDIs. None of the potential participants who were contacted refused to participate.

**TABLE 1:** Demographic characteristics of the participants

Qualitative method	Participant characteristics	Total participants *n*
Focus group discussion (faculty working in medical colleges)	Professors/Associate Professors (males: 15; females: 8) Assistant Professors/Senior Residents (males: 20, females: 9)	52
In-depth interviews	Internal Medicine specialists (males: 6; females: 0) Pulmonology specialists (males: 4; females: 2) Paediatric specialists (males: 6; females: 0) General practitioners (males: 5; females: 1) Hospital administrators (males: 2; females: 0)	26

The FGDs and IDIs were conducted between January 2018 and July 2019 by ADM, who is a faculty member of the Community Medicine Department at a private medical school holding the qualifications of MBBS and MD (Doctor of Medicine) using an interview guide (Table 2) until a saturation point was attained, and no new information was forthcoming. Reflexivity was applied during data collection and analysis. The interview guide was pilot tested before actual use. ADM has been trained in qualitative methods and has published qualitative research previously. Written informed consent was obtained prior to the interview. The FGDs lasted an average of 50 min, and each IDI lasted approximately 25 min and were conducted in English, were audio recorded and later transcribed. The FGDs/IDIs were held at the participants’ workplace. The interviewer ensured that the interaction took place with prior appointment in a quiet place.

**TABLE 2:** Topics included in the interview guide for conducting focus group discussions and in-depth interviews

Sl. no	Topics*
1	What has been your experience in participating with NTEP? Could you describe both positive experiences and negative experiences?
2	What do you think are the factors that are preventing and enabling the participation of private doctors in NTEP?
3	In what ways can the NTEP increase participation of doctors in the private sector?
4	In what way will the change of regimen from intermittent to daily impact the adoption of the NTEP regimen by PPs in India?

* Followed by appropriate follow-up questions and probes.

NTEP = National TB Elimination Programme; PP = private provider.

A framework approach was used for data analysis.^12^ The framework analysis was based on the following three theories: the Social Learning Theory, the Precede/Proceed Model^13^ and the Theory of Learned Behaviour.^14^ Additional themes were identified during the course of data analysis. Three themes derived from the abovementioned theories were retained in the final coding tree (Figure). Thematic content analysis was done to link emerging themes with the codes.

**FIGURE:**
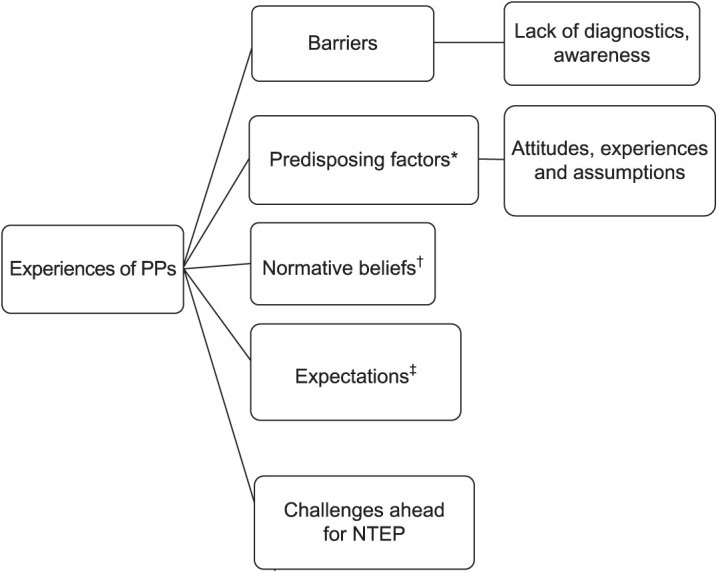
Coding tree depicting the major themes derived from the interviews of PPs. *Predisposing factors derived from the Precede/Proceed Model have been used to collate responses regarding unfavourable attitudes about and unpleasant experiences with NTEP. ^†^Normative beliefs derived from the Theory of Planned Behaviour cover responses of PPs relating to social pressure from peers and patients. ^‡^Derived from Social Learning Theory. This code represents the expectations of healthcare providers (PPs) regarding how the NTEP would respond once they became involved in the programme. PP = private provider; NTEP = National TB Elimination Programme.

Two coders performed the content analysis. A licensed version of NVivo Pro v12 (Lumivero, Denver, CO, USA) was used for qualitative data analysis and management. The COREQ (Consolidated criteria for reporting qualitative research) criteria were adopted for presenting the qualitative results of the study.^15^ Data credibility was ensured by respondent validation. Two specialists who participated in the FGDs were selected randomly. These selected participants were given a draft of the report of this study for their critical comments. The comments obtained were integrated into the analysis and interpretation. An audit trail was maintained to ensure confirmability. Written informed consent was obtained from participants of all the above studies prior to data collection.

Ethical approval was obtained from the institutional review board of the Academy of Medical Sciences, Pariyaram, Kerala, India (letter reference No. G1.2747/12/ACME) where the author was working at the time of data collection.

## RESULTS

Study findings are presented under the following themes (Table 3).

**TABLE 3:** Organisation of results by broad themes and sub-themes

Sl. no.	Main themes	Sub-themes
1	Barriers in participating in NTEP	Expanded array of diagnostics not available under NTEP Need for flexibility in the NTEP Lack of awareness about where NTEP services are available Clarity needed on what NTEP expects from PPs
2	Facilitators in participating in NTEP	Paediatrician-friendly guidelines for paediatric TB Availability of TB drugs in weight bands for paediatric patients Assured supply of full course of anti-TB medication
3	Beliefs and attitudes concerning NTEP	Normative beliefs held by the PPs Predisposing factors: ° NTEP is a way of getting rid of problematic patients ° NTEP is for patients who cannot afford to buy medication Attitudes and experiences related to the NTEP Expectations of PPs once they involve themselves in the NTEP
4	Challenges ahead for the NTEP	The quality of TB care was not perceived to be uniform across the various healthcare levels The presence of a pulmonologist at all centres where TB treatment is provided was thought to be essential Improved quality of interaction between the NTEP staff and patients Setting up of NTEP hubs in private hospitals with NTEP trained medical officers was suggested for the dissemination of guidelines and smooth referral

NTEP = National TB Elimination Programme; PP = private provider.

### Barriers to participating in the NTEP

*Limited range of diagnostics under the NTEP*

Many PPs felt that diagnosis of extrapulmonary TB was relatively complicated and required special investigations which were not available under the NTEP.

…Patients really can’t afford all of those diagnosis because they do not have money, for example Pott’s spine may require an MRI which is not there in NTEP for free… (Internal Medicine specialist, private medical college)

*Need for flexibility in the NTEP*

Flexibility issues identified included 1) the inability to change the regimen when the patient experienced side effects, 2) difficulty in processing samples on holidays, 3) formalities involved in extending the regimen if required, 4) inability to individualise regimens, and 5) challenges faced by patients in medication collection and intake under DOT provider supervision (as reported by patients to PPs. One provider found it difficult to start their patients on empirical anti-TB treatment in the absence of a tissue diagnosis:

…If I say there is an unexplained bone marrow suppression, … and I want to take an empirical TB course, they (NTEP) will not budge. (Private pulmonologist)

*Lack of knowledge about the location of NTEP facilities*

Many PPs initially struggled to contact the NTEP for patient referrals:

…you have no way of accessing the nearest TB officer or DHO (District Health Officer) who can guide you and say, this is your nodal centre. (Private pulmonologist)

*Lack of clarity on what the NTEP expects from PPs*

The PPs were unsure about the scope of their participation in the NTEP:

What do you mean by participation? Participation may be just notification … I have a case, how is he/she diagnosing it, treating it? (Pulmonology specialist, private medical college)

*Facilitators of participating in the NTEP*

All private sector paediatricians acknowledged that the newer guidelines for paediatric TB were more user-friendly and expressed satisfaction with the availability of TB drugs in weight bands. Furthermore, the guaranteed supply of full course of anti-TB medication under the NTEP was appreciated by all the PPs.

### Beliefs and attitudes about the NTEP

*Normative belief held by PPs*

There was a general perception that patients expected prompt laboratory investigation results. Most PPs believed that request for a quick diagnostic test was possible in the private sector laboratories, but not at NTEP diagnostic centres:

…And in private practice, at least, the expectation of the patient is quite high. So, if I say suspected TB and if I say sputum AFB smear, they are asking me whether I will see them that evening only [get the report] (Private pulmonologist).

*NTEP as a way of getting rid of problematic patients*

Some PPs considered treating TB as being problematic, especially if it involved dealing with patients who defaulted, and were more likely to report such cases.

… To get rid of that headache, we refer. Let them (the NTEP) do the follow-up. (Internal Medicine specialist, private medical college)

*NTEP is for patients who cannot afford to buy medication*

One of the most common preconceptions about the NTEP was that it was meant for patients who could not afford to buy anti-TB drugs from private pharmacies, and that patients who could afford medicines should be given a choice.

We give them a choice whether you can afford for 9–12 months treatment or not. Some people who cannot afford, we usually refer them to Government Chest Hospital TB hospital. (Private Internal Medicine specialist)

*Patient feedback on the NTEP*

PP satisfaction with the NTEP depended on feedback from patients. Patients were found to be initially hesitant about availing free services as they were sceptical about the quality of such services. A general practitioner had this to say about referring to the NTEP:

…Now if you ask us to direct all the diagnosed cases to them (NTEP), all failures will be there. They will never visit their place, they will never take the tablets, and they may suffer… and tuberculosis will spread like anything. (General Practitioner)

PPs seldom received feedback on patients they had referred, which acted as a deterrent to further referrals to the NTEP. Although the NTEP has been using the daily regimen for several years, specialist PPs felt it had been introduced too late. Another common belief about the NTEP was that the patients put on treatment were rarely examined clinically.

Almost every participant PP sought more frequent opportunities for dialogue with the NTEP. However, interaction between the PPs and the NTEP was usually unpleasant, with unjust apportionment of blame on PPs. One PP questioned the logic of referring a patient to the NTEP if there was nothing to be gained from it:

…To make any person participate in something, they will see what I gain in this that is basic human mentality. So, I don’t know what the gain is to a private practitioner… (Internal Medicine specialist, private medical college)

Patients sometimes refused to go to a NTEP centre because of the stigma attached to TB and requested PPs to keep their details confidential:

### Expectations of PPs once they involve themselves in the NTEP

Many of the PPs who were not associated with a medical college felt that more attention was being given to PPs in medical colleges and wanted one-on-one training:

To tell you the truth, I would prefer it one to one and I would not mind spending a few hours learning it and I think it is more important. In a group, I think you tend to lose concentration… (Private General Practitioner).

Based on feedback from patients, study PPs suggested three strategies that could save patient travel time: 1) home treatment for patients with TB, rather than at a health centre/DOTS providers’ home, 2) the establishing of more health centres, and 3) accreditation of private laboratories by the NTEP.

### Challenges ahead for the NTEP

The quality of TB care was not perceived to be uniform across the healthcare system. Tertiary-level care tended to be better than at care offered at the lower levels.

If I send the patient to the key nodal centre for the state, the care tends to be quite good. But if they go to the secondary *taluk* [an administrative district for taxation purposes, typically comprising a number of villages] TB hospital, what is done, how they approach is not uniformly done. (Private pulmonologist)

The presence of a pulmonologist at all centres where TB treatment is provided was thought to be essential. Screening all contacts for TB and drug susceptibility testing at the point of care were also suggested. Treating patients weighing less than 40 kg and more than 60 kg using the adult fixed-drug combination (FDC) dosage was felt to be inappropriate, and weight band-based FDCs for these groups was recommended. Most of the PPs felt uneasy about the fact that anti-TB drugs were available in private pharmacies. Another recommendation was to enhance the quality of the interaction between the NTEP staff and patients. Setting up NTEP hubs in private hospitals with NTEP-trained medical officers was suggested for the dissemination of guidelines and a smoother referral process.

## DISCUSSION

The present study set out to elicit experiences of, barriers to and facilitators of PP participation in the NTEP. The non-availability of a comprehensive range of diagnostics and lack of flexibility in the NTEP were barriers to participation in NTEP. Some common PP preconceptions included the belief that NTEP treatment was meant for those who did not have the means to purchase medications. Attitudes and previous experiences with the NTEP made PPs sceptical about the NTEP regimen, although the transition from the intermittent regimen to the daily regimen was widely welcomed. Although more frequent interactions with the NTEP were sought, some expressed bitterness about previous interactions.

In a public–private model implemented in Chennai, India, the self-identified needs of PPs were addressed. This resulted in a high provider participation and substantial contribution to TB case notifications.^16^ Another study aimed at costing scale-up of private sector engagement in TB care in three cities in India found the costs incurred to be similar to the costs incurred in the public sector.^17^ Private sector engagement in TB control in Indian cities was found to be cost-effective, particularly when it was specific to local settings.^18^

Lack of flexibility in the NTEP guidelines was one of the barriers to private sector involvement in the NTEP in Kerala, India.^10^ In an investigation into PP viewpoints regarding their role in TB control in India, the absence of flexibility in prescribing anti-TB medications was identified as a hindrance to their participation in the national TB program.^19^ Rigid work timings was an important concern raised by PPs as a deterrent to involvement in the NTEP in Southern India.^20^ The inability to take independent decisions despite disagreement with clinical guidelines is known to decrease the use of these guidelines in daily practice.^15^ This was illustrated by the perception held by PPs concerning patients’ expectations, which discouraged them from referring patients to the NTEP. This reluctance was partly due to inflexible treatment regimens and the low probability of receiving a speedy diagnosis.

There is limited evidence of PPs referring patients to the NTEP, as they perceive TB treatment to be fraught with challenges. However, one study in Southern India came across PPs referring patients to the NTEP as they could not ensure completion of treatment, and also, when it was felt that the patient could not afford diagnostic facilities.^14^ The inability of patients to afford private treatment emerged as the predominant reason for referring them to the NTEP. Several other studies from Southern India^10^,^21^,^22^ and one study from Bali, Indonesia,^23^ have documented PPs referring some patients to the national programme as they felt the patients could not afford private treatment.

Our study findings are in line with the findings from other studies,^9^,^17^,^20^,^24^ which suggest that PPs occasionally had patients who refused to go to the NTEP for treatment—mainly due to concerns about the quality of NTEP treatment. Similar concerns about quality and TB stigma were voiced by PPs in Southern India.^14^ Although the daily regimen for TB had already been implemented in Bangalore City at the time of the present study, almost all PPs felt this switch had come too late. The belief that a daily regimen is better than an intermittent regimen had been expressed strongly in several studies in South India.^10^,^14^,^15^ Surprisingly, in one qualitative study from Kerala, South India, health workers perceived that patients were more comfortable with the intermittent regimen than the daily regimen due to drug side effects.^9^ Several PPs in the out study expressed unhappiness that patients were only subjected to sputum tests and rarely examined clinically during follow-up. This view was endorsed by a study in Kerala, where PPs were believed to ignore the public health aspects of TB (e.g., sputum positivity indicating infectiousness) and focus only on clinical management.^10^

Other studies have also reported that PPs sometimes do not adhere to guidelines because they consider these erroneous.^25^ Studies from Southern India have shown that PPs considered the NTEP guidelines as being primitive and that regular dissemination of NTEP guidelines to the PPs would be necessary for them to implement it in their daily practice.^14^ Mathew et al. showed that PPs were not well informed about changes in NTEP guidelines, especially since the guidelines changed frequently.^9^

In a qualitative investigation into TB treatment delivery, the accessibility and quality of TB treatment emerged as crucial factors influencing patients’ choices of treatment sources.^23^ In this study, some PPs expressed concerns about the accessibility of NTEP services and suggested strategies that could increase accessibility. Some PPs even found locating the nearest NTEP facility problematic. However, the quality of TB care has consistently been a fundamental principle guiding the efforts of the NTEP.^26^

### Strengths and limitations

Opinions and beliefs expressed by the providers present a comprehensive view of the NTEP-PP interaction landscape. The applicability of the findings may be restricted to other urban conglomerations in India with comparable contexts. As only two hospital administrators were interviewed, theoretical saturation of the opinions of this subset of PPs may not have been achieved.

## CONCLUSIONS

Challenges identified by PPs include improvement of the quality of TB care provided by the NTEP, especially at the lower levels of care, availability of a comprehensive range of diagnostics, greater flexibility and user-friendliness for both PPs and patients, fostering more frequent interactions with PPs for the dissemination of updates and changes and promoting more compassionate interactions with patients at NTEP centres.
